# PRRSV infection induces mitochondrial damage and releases mitochondrial dsRNA to activate the NLRP3-dependent inflammatory response

**DOI:** 10.1186/s13567-026-01854-3

**Published:** 2026-10-11

**Authors:** Jiangnan Li, Caihong Bi, Liguo Gao, Zengyu Shao, Li Huang, Changjiang Weng, Hongyang Liu

**Affiliations:** 1 Division of Fundamental Immunology, State Key Laboratory of Animal Disease Control and Prevention, Harbin Veterinary Research Institute of Chinese Academy of Agricultural Sciences, Harbin, 150069 China; 2 Heilongjiang Provincial Key Laboratory of Veterinary Immunology, Harbin, 150069 China

**Keywords:** PRRSV, mtdsRNA, NLRP3, ASC, IL-1β, nsp11, mitochondrial DAMP, inflammation

## Abstract

Porcine reproductive and respiratory syndrome (PRRS), caused by the PRRS virus (PRRSV), remains one of the most economically significant infectious diseases affecting the global swine industry, resulting in annual losses exceeding US $1.2 billion in the USA alone. PRRSV infects porcine alveolar macrophages (PAMs) and induces profound mitochondrial dysfunction, leading to the release of mitochondrial DNA (mtDNA). However, the mechanisms by which PRRSV-induced mitochondrial damage acts as a damage-associated molecular pattern (DAMP) to trigger inflammatory responses have not been fully elucidated. Here, we report that highly pathogenic PRRSV (HP-PRRSV) infection upregulates interleukin-1β (IL-1β) messenger RNA (mRNA) expression and protein secretion in a dose- and time-dependent manner. Transmission electron microscopy revealed progressive mitochondrial swelling, cristae disorganization, and matrix loss during HP-PRRSV infection. Using RNAscope combined with MitoGreen staining, we discovered that mitochondrial double-stranded RNA (mtdsRNA), generated from bidirectional transcription of mitochondrial DNA, is released into the cytosol during PRRSV infection. In vitro, transcription-driven mtdsRNA or mtdsRNA isolated from PAMs directly activated the NLRP3 inflammasome, inducing caspase-1 cleavage and IL-1β secretion. Mechanistically, the PRRSV-encoded nonstructural protein 11 (nsp11) inhibited NLRP3-dependent inflammatory responses by suppressing the oligomerization of the apoptosis-associated speck-like protein containing a caspase recruitment domain (CARD) (ASC), thereby constraining excessive inflammation and establishing a delicate balance between pro-inflammatory signaling and viral immune evasion. Collectively, our findings identify mtdsRNA as a previously unrecognized mitochondrial DAMP that links PRRSV-induced mitochondrial injury to NLRP3 inflammasome activation, while also revealing a counter-regulatory role for nsp11 in modulating inflammation.

## Introduction

Porcine reproductive and respiratory syndrome virus (PRRSV) infection causes severe respiratory disease in pigs of all ages and reproductive failure in pregnant sows, a syndrome collectively known as porcine reproductive and respiratory syndrome (PRRS) [1, 2]. PRRSV is an enveloped, positive-sense RNA virus belonging to the order Nidovirales, family *Arteriviridae* [3–5]. The viral genome consists of 5′ and 3′ untranslated regions (UTRs) flanking multiple open reading frames (ORFs), including *ORF1a*, *ORF1b*, *ORF2a*, *ORF2b*, *ORFs 3–5*, *ORF5a*, *ORF6*, and *ORF7* [3, 4]. *ORF1a* and *ORF1b* occupy approximately three-quarters of the genome and encode 14 nonstructural proteins (nsp1α, nsp1β, nsp2-12) essential for viral replication [3, 5]. The economic toll of PRRSV is staggering: recent analyses estimate annual losses of US $1.2 billion in the USA swine industry alone, with worldwide losses exceeding US $2.5 billion per year [2, 6]. Vaccination remains the primary preventive measure; however, the remarkable genetic plasticity of PRRSV, particularly among circulating strains, has rendered many commercial modified live vaccines (MLVs) only partially effective [7]. Recent evidence even suggests that MLV vaccination can exert evolutionary selection pressure, driving viral diversification [7]. Novel experimental antivirals, including the endoribonuclease inhibitor A8-A2 targeting nsp11, are under development but are not yet clinically available [8].

PRRSV infection induces a robust secretion of inflammatory cytokines, including interleukin-1β (IL-1β), interleukin-6 (IL-6), and tumor necrosis factor-alpha (TNF-α), in infected piglets [9, 10]. Primary porcine alveolar macrophages (PAMs)—the main cellular targets of PRRSV in vivo [11, 12]—respond to infection by upregulating proinflammatory gene expression and activating multiple innate immune pathways. Previous studies have shown that PRRSV infection activates toll-like receptor 4/myeloid differentiation primary response 88 (TLR4/MyD88) signaling, enhancing pro-IL-1β expression via nuclear factor kappa B (NF-κB), while simultaneously triggering NLRP3 inflammasome assembly, which leads to IL-1β maturation and secretion [12, 13]. However, the mechanisms linking viral replication to NLRP3 activation, as well as the regulatory strategies that fine-tune this response, remain incompletely understood.

Mitochondria are increasingly recognized as central hubs in innate immunity [14, 15]. Beyond their canonical roles in energy metabolism, these organelles function as signaling platforms for antiviral responses and as sources of damage-associated molecular patterns (DAMPs) [16, 17]. During cellular stress, mitochondrial components—including reactive oxygen species (ROS), mitochondrial DNA (mtDNA), and, more recently, mitochondrial double-stranded RNA (mtdsRNA)—can leak into the cytoplasm and activate pattern recognition receptors (PRRs), thereby triggering inflammatory cascades [18–20]. MtdsRNA, which arises from symmetrical bidirectional transcription of the heavy and light strands of mitochondrial DNA, structurally resembles viral double-stranded RNA (dsRNA) and is thus particularly prone to activating immune sensors [20, 21]. Recent studies have identified SRA stem-loop interacting RNA-binding protein (SLIRP) as a key regulator that stabilizes mtdsRNA and amplifies antiviral signaling through positive feedback. Aberrant accumulation of mtdsRNA has been linked to autoimmune diseases, degenerative disorders, and viral pathogeneses [22]. However, the role of mtdsRNA in PRRSV pathogenesis and whether viral infection triggers its release to activate inflammasomes remain unexplored.

Several recent advances have enhanced our understanding of PRRSV–mitochondrial interactions during infection. Transcriptomic and metabolic profiling studies using Seahorse technology have demonstrated that PRRSV-2 infection of PAMs downregulates genes involved in oxidative phosphorylation and the electron transport chain, resulting in impaired mitochondrial respiration and reduced mitochondrial fitness early in infection [23]. Notably, although PRRSV-induced mitochondrial dysfunction is well established [24, 25], recent evidence suggests that significant ROS elevation may not be universally observed across all strains or cell types, indicating context-dependent mechanisms [1, 2]. Additionally, beyond the direct release of mitochondrial nucleic acids, PRRSV has been shown to trigger calcium-mediated mitochondrial fission and PINK1-Parkin-dependent mitophagy, which redirects cellular metabolism toward glycolysis to support viral replication [25]. Collectively, these findings indicate that PRRSV actively remodels mitochondrial dynamics and function; however, how these alterations contribute to inflammatory signaling remains to be fully elucidated.

PRRSV-encoded nonstructural proteins play multifaceted roles in modulating host immunity. A systematic screen using a porcine iGLuc reporter system identified three nsps (nsp1β, nsp2, nsp5) that activate the NLRP3 inflammasome, while four nsps (nsp1α, nsp7, nsp10, nsp11) inhibit its activation, with nsp10 and nsp11 exhibiting the most potent inhibitory effects [26]. Notably, nsp2 has recently been shown to activate the NLRP3 inflammasome through an IKKβ-dependent mechanism that promotes NLRP3 translocation to the dispersed trans-Golgi network, where oligomerization occurs [27]. Even more strikingly, PRRSV nsp5 has been identified as a central mediator of immune imbalance: it recruits NLRP3 to the endoplasmic reticulum (ER)–mitochondrial interface, triggering ER stress and Ca^2+^ leakage that activate NLRP3, while simultaneously retaining stimulator of interferon genes (STING) in the ER to suppress type I interferon induction, thereby driving the characteristic “high inflammation, low interferon” phenotype of PRRSV infection [28]. Nsp11, which possesses endoribonuclease (NendoU) activity essential for viral replication, has been reported to inhibit NLRP3-dependent IL-1β production, although the precise molecular mechanism remains unclear [29]. Intriguingly, recent work has revealed that nsp11 undergoes K48-linked polyubiquitination at the catalytic residue K173, mediated by the host E3 ligase TRIM29, which targets nsp11 for proteasomal degradation, thereby counteracting its immunosuppressive effects [30]. This ubiquitination mechanism is evolutionarily conserved across most arteriviruses, suggesting a fundamental host strategy to counteract nsp11-mediated immune evasion [30]. Targeting nsp11 also holds therapeutic promise: small molecules such as A8-A2 that inhibit NendoU activity have been shown to block PRRSV replication and reverse nsp11-mediated suppression of interferon production in PAMs [8].

In this study, we investigated whether PRRSV-induced mitochondrial damage leads to the release of mtdsRNA and whether this released mtdsRNA contributes to NLRP3 inflammasome activation. Furthermore, we explored the regulatory role of nsp11 in controlling excessive inflammation during PRRSV infection, situating these findings within the rapidly evolving understanding of PRRSV immunopathogenesis, mitochondrial DAMP biology, and therapeutic strategies targeting viral immune evasion.

## Materials and methods

### Antibodies and plasmids

Rabbit anti-nsp11 polyclonal antibody (pAb) was provided by Wenhai Feng, professor at China Agricultural University. Mouse anti-N protein monoclonal antibody (mAb) was donated by Xi Li, professor at the Harbin Veterinary Research Institute, Chinese Academy of Agricultural Sciences. Rabbit anti-HA mAb and anti-Flag mAb were purchased from Cell Signaling Technology. Mouse anti-GAPDH mAb was purchased from Proteintech. Plasmids expressing hemagglutinin (HA)-tagged swine NLRP3, ASC, pro-Caspase-1, and pro-IL-1β and Flag-tagged nonstructural proteins (nsps) of PRRSV, have been previously described.

### Cells and viruses

PAMs were isolated from the bronchoalveolar lavage fluid of 30-day-old pigs that tested negative for PRRSV. The PAMs were cultured in a specialized growth medium at 37 °C in a 5% CO_2_ incubator. HEK293T cells were maintained in standard Dulbecco’s Modified Eagle Medium (DMEM) supplemented with 10% heat-inactivated fetal bovine serum (FBS) under the same incubation conditions. The HP-PRRSV HuN4 strain was provided by Professor Zhijun Tian from the Harbin Veterinary Research Institute, Chinese Academy of Agricultural Sciences. Rabbit anti-NLRP3 polyclonal antibody (pAb), anti-ASC pAb, anti-Caspase-1 p20 pAb, and anti-IL-1β pAb were generated in our laboratory as previously described.

### Enzyme-linked immunosorbent assay (ELISA)

The levels of secreted IL-1β and TNF-α in the cell culture supernatants were measured using commercially available porcine IL-1β and porcine TNF-α ELISA kits (R&D Systems) according to the manufacturer’s instructions.

### RNA extraction and quantitative real-time polymerase chain reaction (qRT-PCR)

PAMs, either infected or uninfected with PRRSV, were collected and centrifuged, after which the supernatants were discarded. The cells were washed with phosphate-buffered saline (PBS) and then lysed using 1 mL of TRIzol reagent. Total RNA was extracted from the collected cells following the manufacturer’s instructions (Invitrogen). The RNA concentration was adjusted to 50 ng/µL, and reverse transcription was performed using the PrimeScript^TM^ RT Master Mix (Perfect Real Time) kit (Takara). The resulting cDNA served as a template and was mixed with TransStart^®^ Top Green qPCR SuperMix reagent (TransGen Biotech). The reaction mixture consisted of 1 µL of template, 0.4 µL of forward primer (10 µM), 0.4 µL of reverse primer (10 µM), 10 µL of 2 × TransStart^®^ Top Green quantitative polymerase chain reaction (qPCR) SuperMix, and 8.2 µL of nuclease-free water. After thorough mixing, the samples were analyzed using an Agilent Mx3005P fluorescence quantitative PCR instrument. The thermal cycling protocol for the melting curve analysis was as follows: 94 °C for 30 s; 40 cycles of 94 °C for 5 s and 60 °C for 30 s; 95 °C for 5 s; 68 °C for 59 s; 72 °C for 30 s; and 55 °C for 30 s. The primers used for qRT-PCR in this study are listed in Table 1.

**Table 1:** **The primers used for qRT-PCR**

Genes	Primers	Sequences (5’-3’)
IL-1β	Sense	CCCAAAAGTTACCCGAAGAGG
	Antisense	TCTGCTTGAGAGGTGCTGATG
TNF-α	Sense	ACCACGCTCTTCTGCCTACTGC
	Antisense	TCCCTCGGCTTTGACATTGGCTAC
HPRT	Sense	GCCGAGGATTTGGAAAAGG
	Antisense	GCACACAGAGGGCTACGATG

### Western blotting

After experimental treatment for viral infection or transfection, cell lysates were collected. According to the bicinchoninic acid (BCA) Protein Assay Kit (Thermo Fisher) manual, the total protein amounts in each group of samples were adjusted to a consistent value. The lysates were subjected to 12% sodium dodecyl sulfate-polyacrylamide gel electrophoresis (SDS-PAGE), and the proteins on the gel were then transferred to a polyvinylidene difluoride (PVDF) membrane. The PVDF membrane was blocked with 5% skim milk at room temperature for 2 h, followed by incubation with the appropriate primary antibody specific to the target protein overnight at 4 °C. After removing the primary antibody, the membrane was washed with tris-buffered saline with Tween-20 (TBST) three times for 10 min each. Subsequently, the secondary antibody, corresponding to the primary antibody, was added and incubated on a horizontal shaker at room temperature for 1 h. The membrane was washed again with TBST three times for 10 min each. After incubation with an infrared-labeled secondary antibody, the image was scanned using an Odyssey infrared imager (LI-COR Biosciences).

### Cell transfection

Plasmid transfection of HEK293T cells: when the cells in 6-well plates reached 90% confluence, they were transfected using polyethylenimine (PEI) (Sigma-Aldrich). To reconstitute the NLRP3 inflammasome in HEK293T cells in vitro, the cells were transfected with plasmids expressing HA-NLRP3, HA-ASC, HA-Caspase-1, HA-IL-1β, and the target plasmid for 24 h. Cell supernatants were collected and analyzed for mature IL-1βby ELISA, and the cells were lysed for analysis by western blotting.

Plasmid transfection of PAMs: cells were seeded in 24-well plates, and the medium was changed after 4 h. The transfection procedure was performed overnight using X-tremeGENE HP DNA Transfection Reagent (Roche) according to the manufacturer’s instructions. Cells were collected for subsequent experiments 24 h.

RNA transfection of PAMs: PAMs were evenly distributed into 24-well plates and transfected using the TransMessenger Transfer Reagent kit (Qiagen) after overnight culture. Following 3 h of transfection, the transfection solution was removed, the cells were rinsed with PBS, and fresh culture medium was added.

Small interfering RNA (siRNA) transfection of PAMs: small interfering RNAs (siRNAs) targeting the *NLRP3* gene (siNLRP3) or a nonspecific control siRNA (siNC) were transfected using Lipofectamine^®^ RNAiMAX Reagent (Invitrogen) following the manufacturer’s instructions. The efficiency of NLRP3 protein knockdown was evaluated by western blotting.

### Electron microscopic examination

PAMs were plated on 10 cm dishes, and cells were collected at various time points following PRRSV infection, with the culture medium discarded after each collection. The cells were transferred to 1.5 mL Eppendorf tubes and centrifuged at 3000 rpm for 15 min. The supernatants were then discarded, and glutaraldehyde was added for fixation. The samples were subsequently sent to the electron microscopy facility for transmission electron microscopy analysis.

### RNAscope

RNAscope was performed using the RNAscope^®^ Multiplex Fluorescent Reagent Kit version 2 Assay (Advanced Cell Diagnostics) according to the manufacturer’s instructions.

### Mitochondrial marker

MitoGreen was diluted to 100 nM in Dulbecco’s modified eagle medium (DMEM) and preheated to 37 °C. When the cells reached an appropriate density, the culture medium was discarded, and the cells were collected. The cells were then washed with PBS and fixed with 4% paraformaldehyde for 30 min. After washing the cells again with PBS, the preheated MitoGreen solution (37 °C) was added and incubated at 37 °C for 30 min. Following a final PBS wash, the cells were observed using a laser confocal fluorescence microscope.

### In vitro transcription

Four fragments of the mitochondrial *ND5* gene were selected: ND5-F200 (nt 1–200), ND5-F500 (nt 1–500), ND5-F1000 (nt 1–1000), and ND5-Full (nt 1–1821). These fragments represent different length truncations from the transcription start site of the *ND5* gene. First, total RNA was extracted from PAMs, and complementary DNA (cDNA) was synthesized by reverse transcription. Next, primers specific to ND5-F200, ND5-F500, ND5-F1000, and ND5-Full were designed to amplify these fragments by PCR using the cDNA as a template. The T7 promoter sequence was incorporated into the DNA through this amplification strategy. MtdsRNA was then synthesized using the MEGAscript^TM^ RNAi Kit (Ambion) following the manufacturer’s instructions. The primers used in this study are listed in Table 2.

**Table 2:** **The primers used for PCR**

Genes	Primers	Sequences (5’-3’)
ND5-F200	Sense	TAATACGACTCACTATAGGGAGAATAAACCCATTCGCCTCACT
	Antisense	TAATACGACTCACTATAGGGAGATGTCAGTTTGAAATGATTAT
ND5-F500	Sense	TAATACGACTCACTATAGGGAGAATAAACCCATTCGCCTCACT
	Antisense	TAATACGACTCACTATAGGGAGAGCTGTGTTGGCGTCTGTTCG
ND5-F1000	Sense	TAATACGACTCACTATAGGGAGAATAAACCCATTCGCCTCACT
	Antisense	TAATACGACTCACTATAGGGAGAAAGCGTGCATGCAGATGTGA
ND5-Full	Sense	TAATACGACTCACTATAGGGAGAATAAACCCATTCGCCTCACT
	Antisense	TAATACGACTCACTATAGGGAGATTACTCGTGGTAATTAAATA

### Extraction of PAMs mtdsRNA

Mitochondrial isolation of PAMs was performed using the Mitochondria Isolation Kit for Cultured Cells (Thermo Fisher) according to the manufacturer’s instructions. Mitochondrial RNA was subsequently extracted as described in Sect. 2.4. Nuclease digestion was carried out to remove mitochondrial single-stranded RNA, and mitochondrial double-stranded RNA (mtdsRNA) was purified using the MEGAscript RNAi Kit (Ambion).

### ASC oligomerization

HEK293T cells were cultured in 6-well plates and transfected with plasmids as described in the figure legends. After 24 h later, the cells were collected and lysed in buffer A (1 M Tris–HCl, pH 7.4; 0.25 M EDTA; 2 mM MgCl_2_; 2.5 M NaCl; 10% glycerol) by passing the lysate 30 times through a 25-gauge needle. ASC oligomers were cross-linked using 2 mM disuccinimidyl suberate (DSS) for 30 min at room temperature. The pellets were centrifuged at 5000 × *g* and mixed with SDS-loading buffer for western blot analysis.

### Confocal microscopy

The plasmids expressing HA-NLRP3, GFP-ASC, and Flag-nsp11 were transfected into prepared HEK293T cells using PEI. At 24 h after transfection, the cells were fixed with 4% paraformaldehyde and permeabilized with 0.3% Triton X-100. After washing three times with PBS, the cells were blocked with 10% FBS. Subsequently, the cells were stained with rabbit anti-Flag monoclonal antibody and goat anti-rabbit TRITC-labeled secondary antibody. Finally, the cells were observed using an LSM800 ZEISS confocal laser scanning microscope.

### Speck quantitation assay

HEK293T cells cultured in 48-well plates were transfected with HA-NLRP3, GFP-ASC, and either pCAGGS-Flag-nsp11 or pCAGGS-Flag-Vector plasmids, as described above. At 24 h post-transfection, the number of cells containing GFP-ASC specks was counted in multiple randomly selected fields. The percentage of GFP-ASC speck-positive cells was calculated by dividing the number of speck-positive cells by the total number of transfected cells. At least three independently transfected wells were analyzed per experiment.

### Flow cytometry

HEK293T cells grown in 12-well plates were transfected with plasmids as described in the figure legends. At 24 h after transfection, the cells were trypsinized and washed with PBS. Cells were then suspended in 4% paraformaldehyde. Finally, the cells were analyzed using SONY-SH800S Flow Cytometer.

### Co-immunoprecipitation (Co-IP) assay

To investigate the physical interaction between nsp11 and ASC, HEK293T cells were cultured in 6-well plates and cotransfected with plasmids expressing HA-tagged ASC (HA-ASC) and/or Flag-tagged nsp11 (Flag-nsp11) using Lipofectamine 2000 (Invitrogen) according to the manufacturer’s instructions. At 48 h post-transfection, cells were harvested and lysed in ice-cold immunoprecipitation (IP) lysis buffer containing 20 mM Tris–HCl (pH 7.4), 150 mM NaCl, 1 mM EDTA, 1% NP-40, and a protease inhibitor cocktail (Roche). After centrifugation at 12 000 × *g* for 15 min at 4 °C, the supernatants were collected and incubated overnight at 4 °C with an anti-Flag monoclonal antibody (Sigma, F3165). Protein A/G agarose beads (Santa Cruz Biotechnology) were then added and incubated for an additional 4 h at 4 °C. The beads were washed four times with immunoprecipitation (IP) lysis buffer, and the immunoprecipitated proteins were eluted by boiling in 2 × SDS loading buffer. The samples were subsequently subjected to western blotting with mouse anti-HA (Sigma, H9658) and mouse anti-Flag antibodies to detect the coprecipitated proteins.

### Statistical analysis

All bar graphs represent the mean ± standard deviation of three independent experiments (*n* = 3). Statistical analyses were conducted using Student’s *t*-test with GraphPad Prism 6 software. A *p*-value of less than 0.05 was considered statistically significant. Significance levels are indicated as follows: *p < 0.05; **p < 0.01; ***p < 0.001; ns, not significant.

## Results

### HP-PRRSV induces IL-1β production and NLRP3 inflammasome activation in PAMs

**Figure 1:**
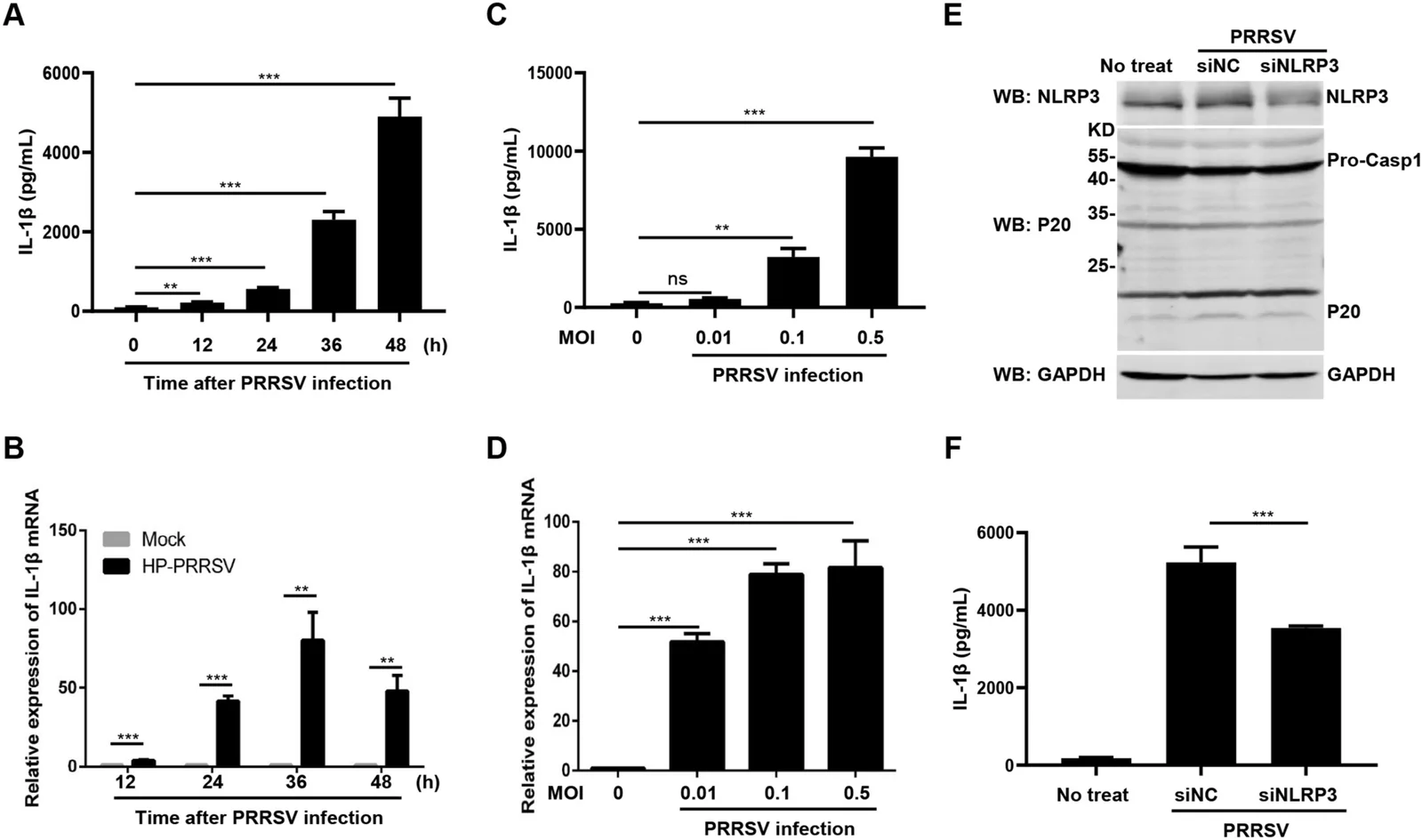
**HP-PRRSV infection enhances NLRP3-dependent inflammasome activation and IL-1β production.** **A** Time course of IL-1β secretion measured by ELISA. **B** Time course of IL-1β mRNA expression measured by qRT-PCR. **C** Dose-dependent IL-1β secretion at 36 hpi. **D** Dose-dependent IL-1β mRNA expression at 36 hpi. **E** Western blot showing NLRP3 knockdown efficiency and reduced p20 production. **F** ELISA confirming decreased IL-1β secretion upon NLRP3 knockdown. Data are presented as mean ± SD. *p < 0.05, **p < 0.01, ***p < 0.001; *ns* not significant.

To characterize the kinetics of IL-1β induction by HP-PRRSV, PAMs were infected with PRRSV, and supernatants and cell lysates were collected at various time points. ELISA and qRT-PCR analyses revealed that IL-1β secretion peaked at 48 h post-infection (hpi) and messenger RNA (mRNA) expression peaked at 36 h; both were significantly upregulated in infected cells (Figure 1A, B). Moreover, the magnitude of IL-1β induction was dose-dependent, with higher viral multiplicities of infection (MOI) eliciting greater responses (Figure 1C, D). To establish that this response requires NLRP3, PAMs were transfected with siRNA targeting NLRP3 prior to infection. Knockdown of NLRP3 markedly reduced both pro-caspase-1 cleavage (assessed by western blotting for the p20 fragment) and IL-1β secretion (Figure 1E, F). These results confirm that HP-PRRSV induces NLRP3-dependent inflammasome activation and IL-1β production in a dose- and time-dependent manner, consistent with previous observations in PAMs [12, 13].

### HP-PRRSV infection triggers progressive mitochondrial damage

**Figure 2:**
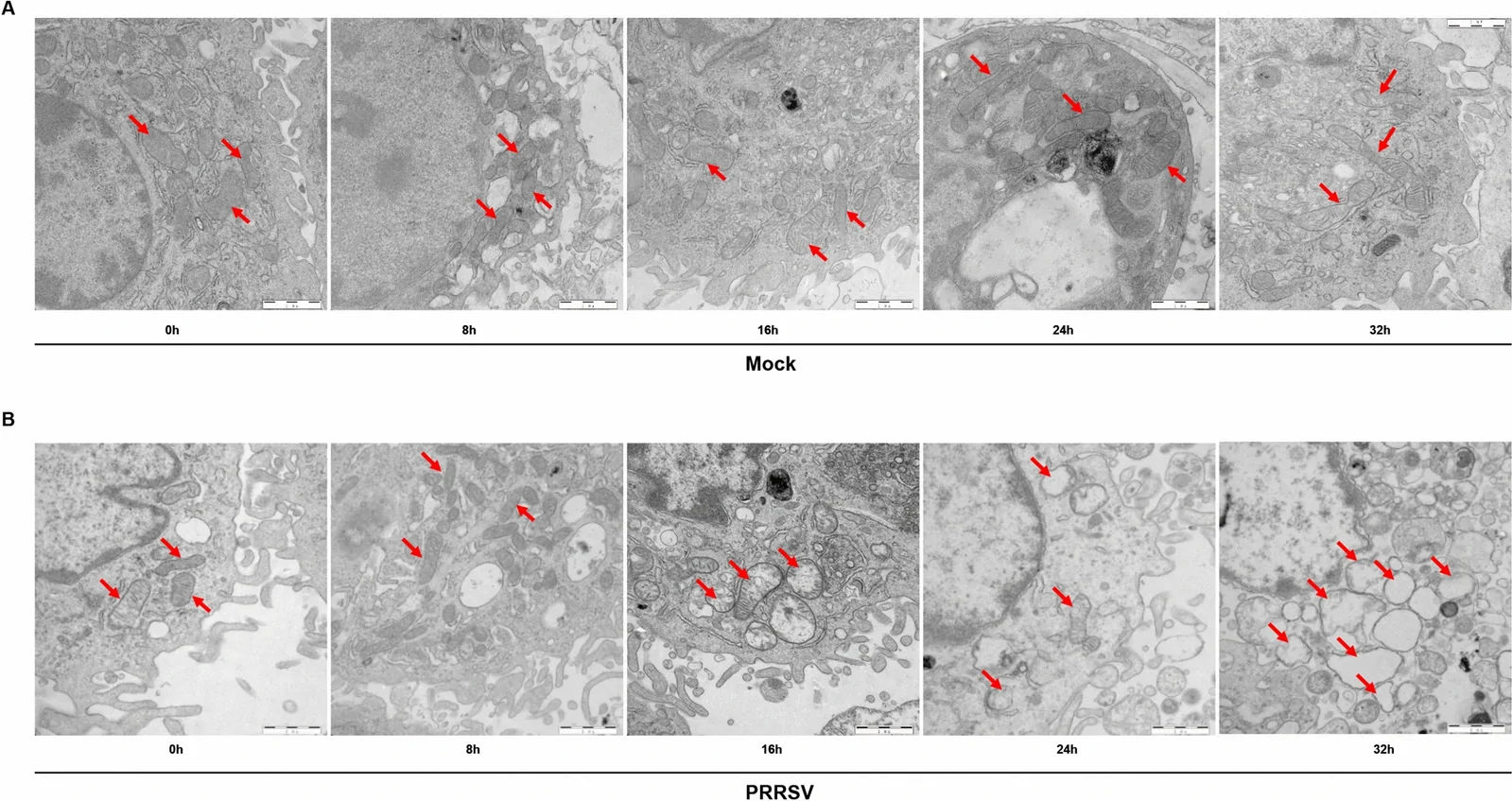
**HP-PRRSV infection causes mitochondrial damage.** **A**, **B** Representative transmission electron microscopy (TEM) images of mitochondria in PAMs from mock-infected (**A**) or PRRSV-infected (**B**) cells at 0, 8, 16, 24, and 32 h post-infection. Red arrows indicate mitochondria. In PRRSV-infected cells, mitochondria exhibited progressive swelling, cristae disruption, and loss of matrix density over time compared with the mock-infected control.

Given that HP‑PRRSV activates the NLRP3 inflammasome and that mitochondria are recognized as important regulators of inflammasome signaling, we next examined whether the virus directly affects mitochondrial integrity at the ultrastructural level. Previous studies have documented that PRRSV infection induces oxidative stress and endoplasmic reticulum dysfunction, which can secondarily impair mitochondrial function [24, 31, 32]. To visually examine the dynamic changes in mitochondrial ultrastructure, transmission electron microscopy (TEM) was performed on PAMs at various time points following PRRSV infection or mock infection. In the mock-infected control cells, mitochondria exhibited a typical elongated morphology with well-defined cristae. In contrast, PRRSV-infected PAMs displayed progressive mitochondrial damage, characterized by marked swelling, cristae disorganization, and reduced matrix density, with the severity of these alterations markedly increasing over the course of infection (Figure 2A, B).

These morphological alterations indicate severe mitochondrial dysfunction. Notably, recent Seahorse-based metabolic analyses have confirmed that PRRSV-2 infection downregulates oxidative phosphorylation and electron transport chain gene expression in PAMs, resulting in reduced maximal respiration and decreased overall mitochondrial fitness [23]. Our electron microscopy findings are consistent with these functional metabolic data, collectively demonstrating that PRRSV induces profound structural and functional mitochondrial impairment in its primary target cells.

### Mitochondrial double-stranded RNA is released into the cytosol during PRRSV infection

**Figure 3:**
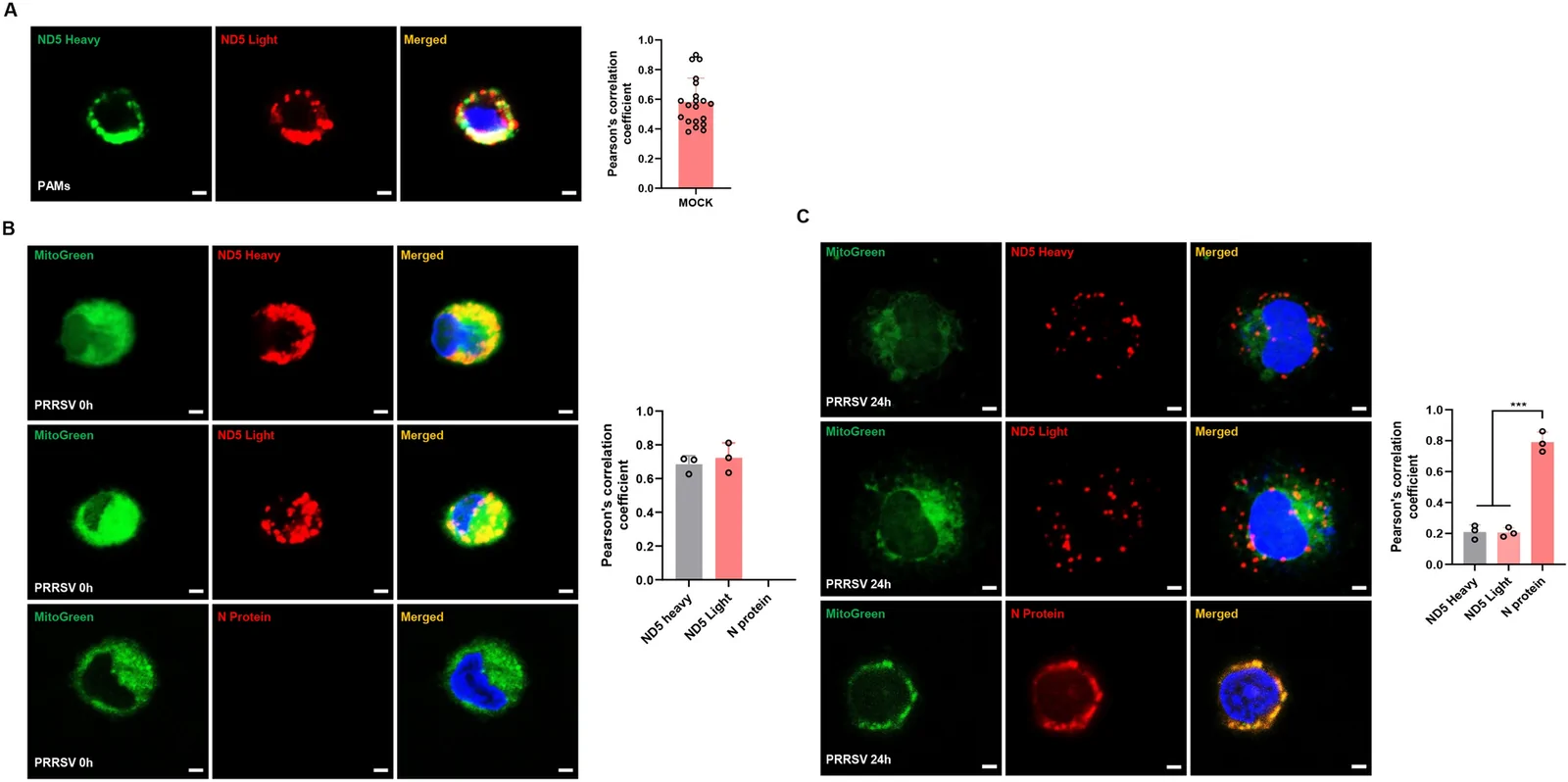
**PRRSV infection causes the release of mtdsRNA into the cytosol.** **A** RNAscope detection of mtdsRNA in uninfected PAMs: heavy-strand RNA (Opal520, green) and light-strand RNA (Opal570, red) colocalize with MitoGreen-stained mitochondria (blue). The bar graph on the right displays the Pearson’s correlation coefficient between the signal intensities of heavy-strand and light-strand RNA. **B** Colocalization analysis in uninfected PAMs confirming the presence of mtdsRNA within mitochondria. The bar graph on the right shows the correlation coefficients of MitoGreen with ND5 Heavy, ND5 Light, and N protein. **C** After PRRSV infection, heavy- and light-strand RNA signals were observed outside mitochondria, indicating mtdsRNA release into the cytoplasm. The bar graph on the right shows the correlation coefficients of MitoGreen with ND5 Heavy, ND5 Light, and N protein.

A key consequence of mitochondrial damage could be the release of immunostimulatory molecules from within the organelle. Because mitochondrial DNA undergoes symmetrical bidirectional transcription, it has the potential to generate mtdsRNA in theory; however, whether PRRSV infection actually triggers mtdsRNA leakage remains unknown. We therefore used RNAscope combined with mitochondrial‑specific staining to track changes in mtdsRNA localization. Mitochondria contain their own genome and, due to the symmetrical bidirectional transcription of the heavy and light strands, generate RNA species capable of forming double-stranded structures [20]. To determine whether mtdsRNA is present in PAMs and whether PRRSV infection induces its release from mitochondria, we performed RNAscope with probes specific for the heavy‑strand and light‑strand transcripts of the mitochondrial *ND5* gene, together with MitoGreen staining to delineate mitochondria. In uninfected PAMs, both heavy‑ and light‑strand *ND5* RNAs were predominantly colocalized with MitoGreen‑labeled mitochondria, confirming that mtdsRNA resides within mitochondria under basal conditions (Figure 3A, B). Upon infection with HP‑PRRSV, however, the colocalization pattern was markedly disrupted. Heavy‑ and light‑strand *ND5* RNA signals were detected outside the mitochondrial compartment, indicating that mtdsRNA is released into the cytosol following viral infection (Figure 3C). These results demonstrate that PRRSV infection triggers the escape of mitochondrial double‑stranded RNA from damaged mitochondria into the cytoplasm, where it may act as a DAMP to engage innate immune sensors. This finding aligns with a rapidly expanding body of evidence identifying mtdsRNA as an immunogenic DAMP that can activate innate immune responses in various pathological contexts [20–22].

### MtdsRNA activates the NLRP3 inflammasome and induces the production of proinflammatory cytokines

**Figure 4:**
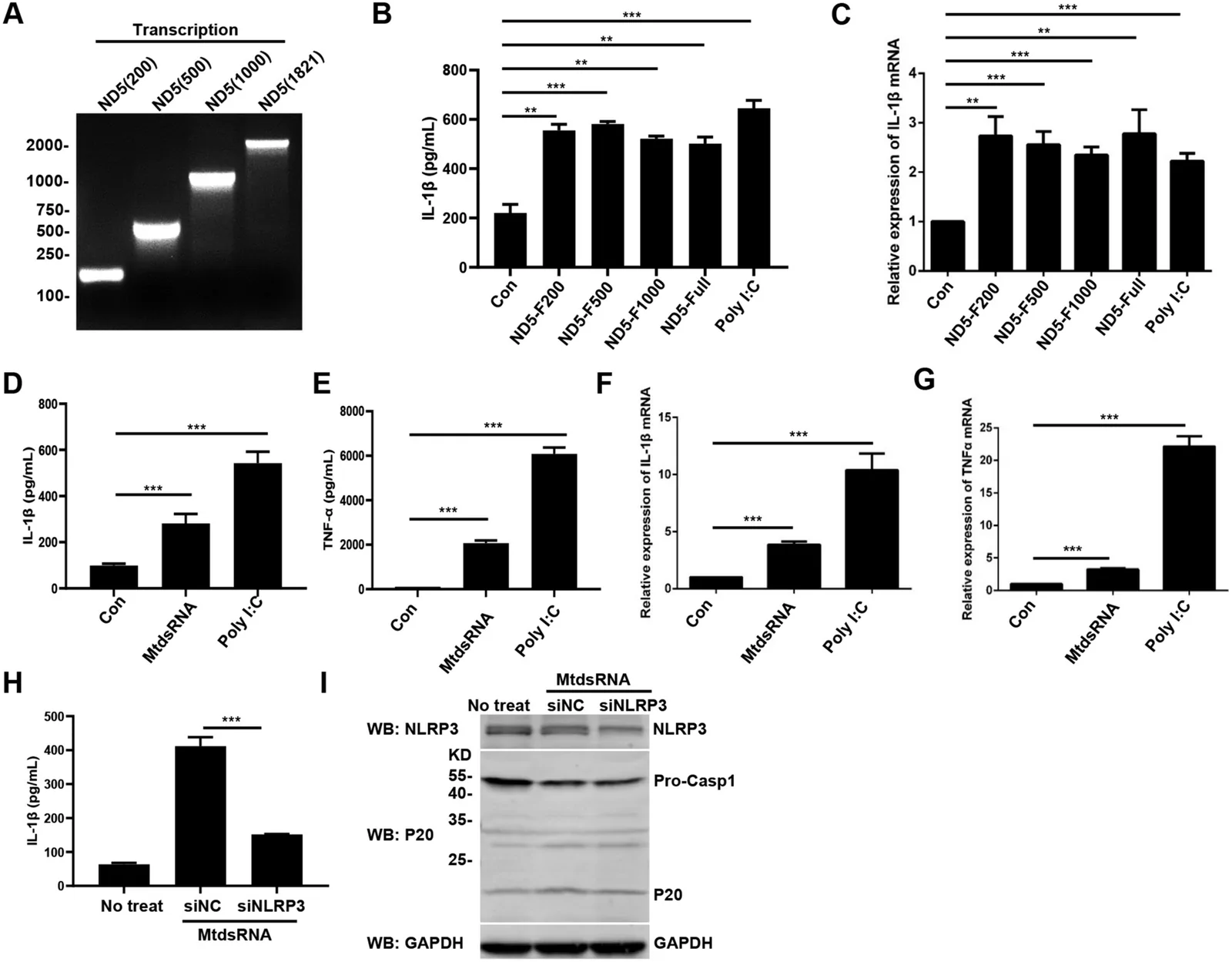
**MtdsRNA activates the NLRP3 inflammasome. A** Schematic representation of synthetic mtdsRNA fragments derived from the *ND5* gene. **B**, **C** Transfection with synthetic mtdsRNA induces secretion of IL-1β as well as IL-1β mRNA expression (**C**). **D**–**G** Endogenous mtdsRNA purified from PAMs also stimulates IL-1β and TNF-α production. **H**, **I** Knockdown of NLRP3 attenuates mtdsRNA-induced IL-1β secretion and pro-caspase-1 cleavage.

Once mtdsRNA is released into the cytosol, a natural question is whether it is functionally active—that is, whether it can actually activate the NLRP3 inflammasome and drive downstream inflammatory responses. To address this, we stimulated PAMs with both synthetic and endogenously purified mtdsRNA and measured the effects on inflammatory cytokine expression. To determine whether mtdsRNA released from mitochondria during PRRSV infection can functionally activate inflammatory responses, we synthesized four mtdsRNA fragments of varying lengths corresponding to the *ND5* gene (ND5-F200; 1–200 bp; ND5-F500: 1–500 bp; ND5-F1000: 1–1000 bp; and ND5-Full: 1–1821 bp) via in vitro transcription (Figure 4A). Transfection of these synthetic mtdsRNA fragments into PAMs robustly induced IL-1β expression at both the mRNA and protein levels, as measured by qRT-PCR and ELISA, respectively (Figure 4B, C). To confirm that endogenously derived mtdsRNA elicits a similar effect, we isolated mitochondria from PAMs, extracted total mitochondrial RNA, and purified native mtdsRNA by digesting single-stranded RNA with nucleases. Transfection of this purified endogenous mtdsRNA also induced IL-1β and TNF-α expression (Figure 4D–G). Importantly, knockdown of NLRP3 using specific siRNA significantly attenuated mtdsRNA-induced pro-caspase-1 cleavage and IL-1β secretion (Figure 4H, I), demonstrating that mtdsRNA activates the NLRP3 inflammasome and requires NLRP3 to promote IL-1β maturation and release. These results establish that mtdsRNA acts as a mitochondrial DAMP capable of triggering NLRP3-dependent inflammatory responses in PAMs. Consistent with this, recent studies have shown that cytosolic mtDNA also activates the NLRP3 inflammasome during PRRSV infection [33], suggesting that multiple mitochondrial nucleic acid species contribute to inflammatory pathology.

### PRRSV nsp11 suppresses NLRP3 inflammasome-dependent IL-1β production

**Figure 5:**
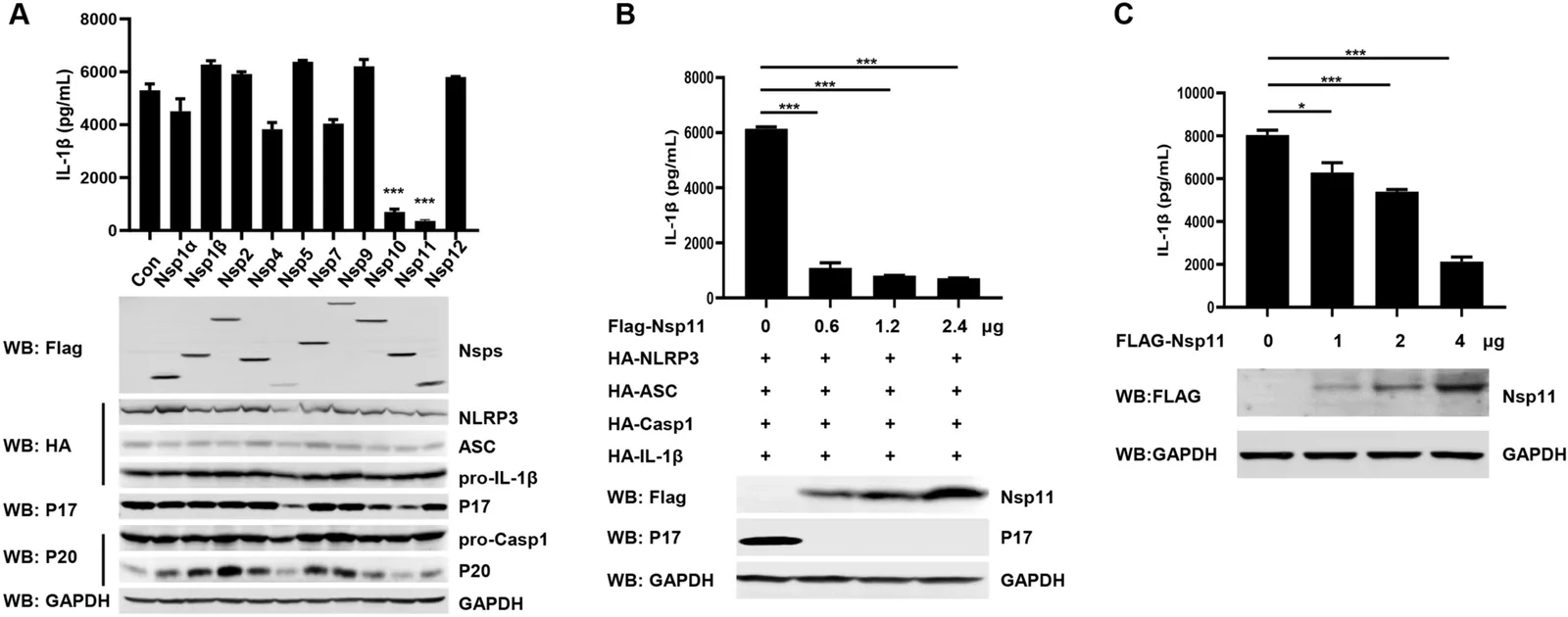
**PRRSV nsp11 inhibits NLRP3 inflammasome-dependent IL-1β production. A** Screening of PRRSV nonstructural proteins (nsps) in the HEK293T NLRP3 inflammasome reconstitution system reveals nsp11 as a potent inhibitor of IL-1β secretion. **B** Dose-dependent inhibition of IL-1β secretion by increasing amounts of nsp11. **C** Overexpression of nsp11 in PAMs suppresses PRRSV-induced IL-1β secretion.

Given that PRRSV infection drives inflammation via mtdsRNA, we wondered whether the virus itself possesses mechanisms to limit this response and prevent excessive immunopathology. Considering that PRRSV encodes multiple nonstructural proteins (nsps), several of which are known to modulate host immunity, we systematically screened all nsps for their effects on NLRP3 inflammasome activity. PRRSV encodes a repertoire of nonstructural proteins that differentially regulate inflammation. To systematically evaluate which nsps modulate NLRP3 inflammasome activity, we reconstituted the NLRP3 inflammasome in HEK293T cells—which lack endogenous NLRP3 components—by cotransfecting expression plasmids for NLRP3, ASC, pro-caspase-1, and pro-IL-1β, along with plasmids encoding individual PRRSV nsps [34]. ELISA analysis of cell culture supernatants revealed that most nsps exerted modest effects on IL-1β secretion. However, nsp11 consistently and potently inhibited IL-1β production, as confirmed by western blotting, which showed reduced processing of pro-caspase-1 to its active p20 form and decreased mature IL-1β (p17) in the presence of nsp11 (Figure 5A). This inhibitory effect was dose-dependent: increasing amounts of nsp11 plasmid (0, 0.6, 1.2, or 2.4 μg) resulted in progressively diminished IL-1β secretion, demonstrating that nsp11 acts as a robust negative regulator of NLRP3 inflammasome activity (Figure 5B). To validate this finding in a more physiologically relevant context, we transfected PAMs with increasing amounts of nsp11 plasmid and then challenged the cells with PRRSV. Consistent with the reconstitution experiments, nsp11 overexpression inhibited PRRSV-induced IL-1β secretion in a dose-dependent manner (Figure 5C). Our results corroborate and extend the screening data, confirming nsp11 as a potent NLRP3 inflammasome inhibitor [26].

It is worth noting that PRRSV deploys multiple nonstructural proteins to modulate host immune responses through complex mechanisms. While nsp11 inhibits NLRP3 activation, other nsps, such as nsp2 and nsp5, activate the inflammasome via distinct mechanisms: nsp2 facilitates NLRP3 translocation to the dispersed trans-Golgi network through an IKKβ-dependent pathway [27], while nsp5 recruits NLRP3 to the ER–mitochondrial interface, triggering activation through ER stress and Ca^2+^ leakage [28]. The interplay between these pro and antiinflammatory nsps likely determines the overall magnitude and duration of the inflammatory response during PRRSV infection.

### Nsp11 inhibits ASC oligomerization to suppress inflammasome activation

**Figure 6:**
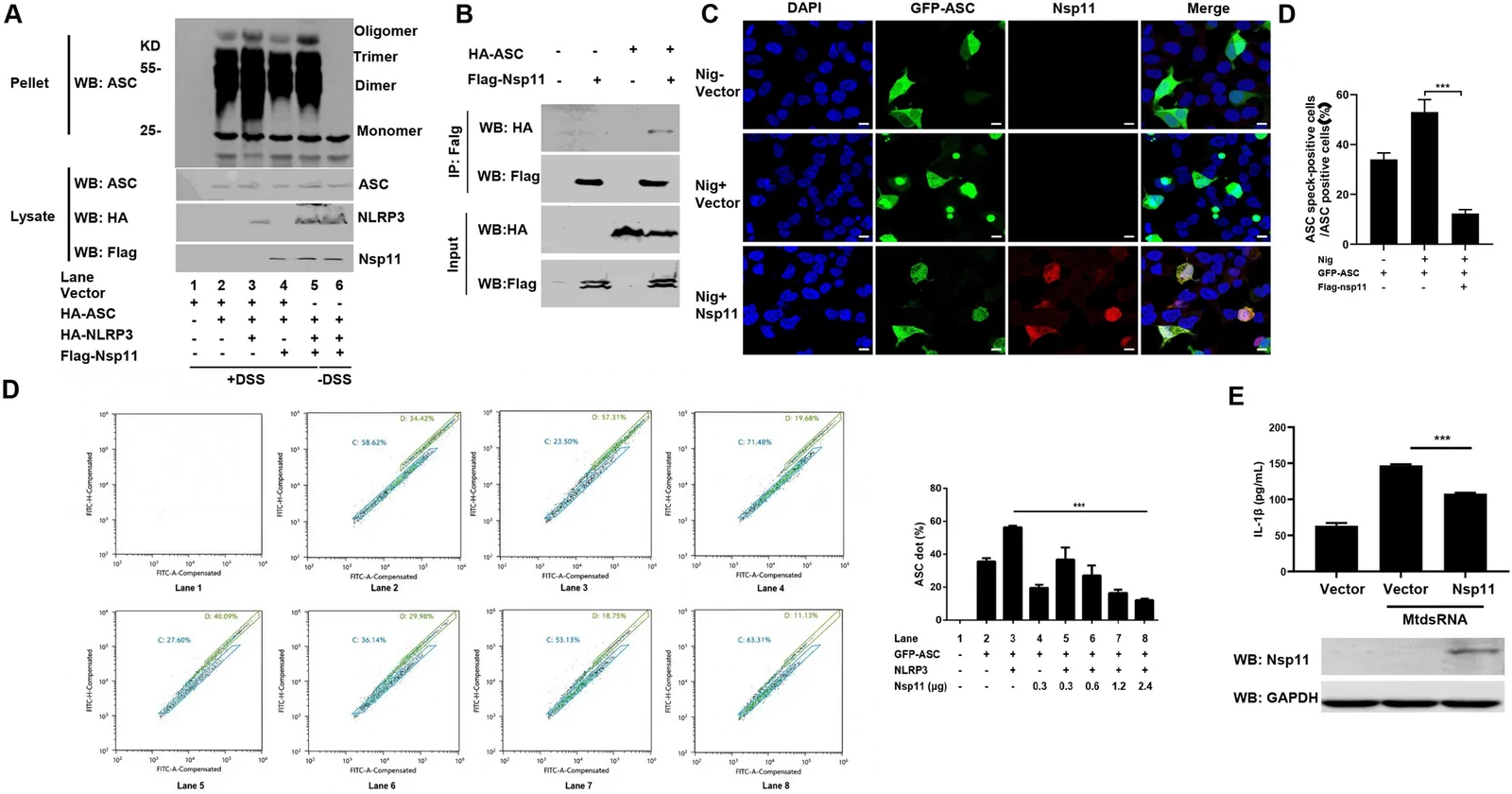
**PRRSV nsp11 reduces inflammation by inhibiting ASC oligomerization**. **A **Lysates were either treated with DSS (+DSS) or left untreated (−DSS), with the −DSS lane serving as a negative control to confirm that the high‑molecular‑weight bands observed in the +DSS lane represent specific cross‑linked ASC oligomers rather than nonspecific aggregates. Western blot analysis further showed that the presence of nsp11 reduced ASC oligomer formation following DSS cross‑linking. **B** HEK293T cells were cotransfected with plasmids encoding HA‑ASC and/or Flag‑nsp11. The resulting cell lysates were subjected to immunoprecipitation with an anti‑Flag antibody, followed by western blot detection with anti‑HA and anti‑Flag antibodies. Whole‑cell lysates were utilized as input controls. **C** Confocal microscopy images of ASC speck formation (GFP-ASC, green) in cells transfected with either empty Flag vector (Vector) or Flag‑nsp11 expression plasmid (Flag‑nsp11, red). Cells were then left untreated or stimulated with 5 µM nigericin (Nig) for 1 h. “Nig +” indicates nigericin stimulation, and “Nig−” indicates vehicle control (DMSO). The transfection groups (Vector versus Flag‑nsp11) are labeled in each panel. **D** Quantification of ASC speck-positive cells. **E** Flow cytometry analysis confirming decreased ASC speck formation. **F** Expression of nsp11 reduces mtdsRNA-induced IL-1β secretion in PAMs.

Although nsp11 clearly suppresses IL‑1β production, its direct target within the inflammasome assembly pathway remains unclear. Since ASC oligomerization is a critical rate‑limiting step in inflammasome formation, we tested whether nsp11 interferes with this process. The adaptor protein ASC plays a central role in inflammasome assembly by oligomerizing in response to upstream activation signals, forming large macromolecular complexes known as ASC specks that serve as platforms for pro-caspase-1 recruitment and activation [18]. The formation of ASC specks is widely regarded as a direct indicator of inflammasome activation. Because nsp11 potently inhibits IL-1β production, we investigated whether it acts by interfering with ASC oligomerization. HEK293T cells were cotransfected with ASC, NLRP3, and either a control vector or nsp11. After 24 h, cells were lysed, and ASC oligomers were cross-linked with DSS and analyzed by western blotting. Nsp11 overexpression markedly reduced the abundance of ASC oligomers compared with control cells (Figure 6A). To provide direct evidence for the molecular mechanism underlying nsp11‑mediated inhibition of ASC oligomerization, we examined the interaction between nsp11 and ASC through Co‑IP assays. As shown in Figure 6B, HA‑ASC was coprecipitated with Flag‑nsp11, indicating that nsp11 directly binds to ASC. Confocal microscopy further revealed that, in the presence of nsp11, upon stimulation with Nigericin (Nig, a well‑established NLRP3 inflammasome agonist), NLRP3^+^ASC^+^ cells exhibited significantly fewer ASC specks compared with control cells (Figure 6C, D). Flow cytometry quantification of ASC speck formation also confirmed the inhibitory effect of nsp11 (Figure 6E). Finally, to directly assess whether nsp11 suppresses mtdsRNA‑driven inflammation, we transfected PAMs with nsp11 and then stimulated the cells with purified mtdsRNA. Nsp11 expression significantly reduced mtdsRNA‑induced IL‑1β secretion (Figure 6F). Collectively, these results indicate that PRRSV nsp11 inhibits NLRP3 inflammasome activation by blocking ASC oligomerization, thereby limiting the assembly of the functional inflammasome complex and attenuating downstream inflammatory cytokine production.

## Discussion

In this study, we elucidate a mechanism by which PRRSV infection triggers NLRP3 inflammasome activation through the release of mtdsRNA from damaged mitochondria, and we also identify nsp11 as a viral factor that suppresses excessive inflammation by inhibiting ASC oligomerization. These findings provide new insights into the interplay between mitochondrial dysfunction and innate immune signaling during *arterivirus* infection, highlighting a sophisticated balance between proinflammatory DAMPs release and viral immune evasion.

**Figure 7:**
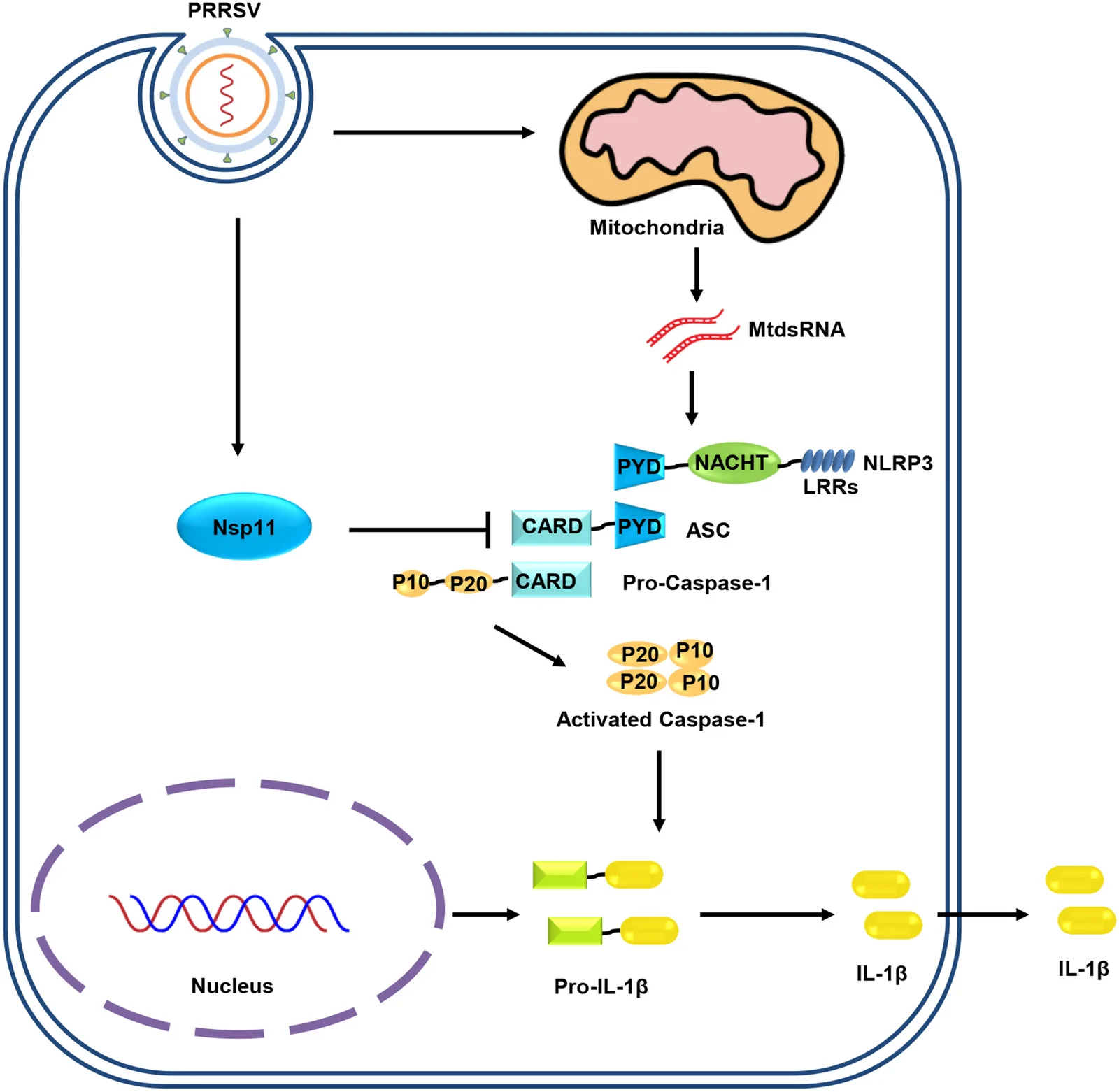
**Schematic model of PRRSV-induced mtdsRNA release and NLRP3 inflammasome activation, with nsp11-mediated regulation.** PRRSV infection induces mitochondrial damage, leading to the release of mtdsRNA into the cytosol. The released mtdsRNA activates the NLRP3 inflammasome, promoting ASC oligomerization, pro-caspase-1 cleavage, and subsequent maturation and secretion of IL-1β. Concurrently, the viral protein nsp11 suppresses excessive inflammation by directly inhibiting ASC oligomerization, representing a viral immune evasion mechanism.

Mitochondria are increasingly recognized as central players in innate immunity [14, 15], serving not only as signaling platforms for antiviral responses but also as reservoirs of DAMPs released upon cellular stress [16, 17]. The concept of mitochondrial DAMPs has expanded considerably in recent years. Initially, reactive oxygen species (ROS) and mtDNA were identified as key activators of the NLRP3 inflammasome [18, 19]. Subsequent studies demonstrated that mtDNA can engage the cGAS-STING and AIM2 pathways, amplifying inflammatory and interferon responses [18, 35]. More recently, mtdsRNA has emerged as a potent immunostimulatory molecule [20, 21]. Structurally resembling viral dsRNA, mtdsRNA can activate cytosolic dsRNA sensors, including RIG-I, MDA5, PKR, and ADAR1 [36–39], eliciting strong type I interferon responses and inflammatory cytokine production [20, 21]. The discovery that SLIRP stabilizes mtdsRNA and amplifies immune signaling through a positive feedback loop underscores the regulatory complexity of this system [22]. Our study contributes to this growing body of knowledge by demonstrating that PRRSV infection promotes the release of mtdsRNA from mitochondria and that this released mtdsRNA activates the NLRP3 inflammasome in PAMs, promoting IL-1β maturation and secretion (Figure 7). To our knowledge, this is the first report specifically identifying mtdsRNA as a mitochondrial DAMP that is released upon PRRSV‑induced mitochondrial damage and triggers NLRP3 inflammasome activation. Although previous work [33] has linked PRRSV‑induced mtDNA release to NLRP3, our study highlights mtdsRNA as a distinct and previously unrecognized mitochondrial nucleic acid species that activates the inflammasome during PRRSV infection.

The finding that PRRSV infection induces time-dependent mitochondrial swelling and cristae damage aligns with ultrastructural observations in other viral infections, including severe acute respiratory syndrome coronavirus 2 (SARS-CoV-2), where viral proteins have been shown to cause mitochondrial fragmentation and dysfunction. However, PRRSV appears to orchestrate mitochondrial remodeling through multiple mechanisms. Beyond inducing direct damage, the virus triggers calcium-mediated mitochondrial fission and PINK1-Parkin-dependent mitophagy, which redirects cellular metabolism to enhance glycolysis and support viral replication [25]. This metabolic reprogramming may influence the inflammatory response by altering the availability of mitochondrial DAMPs. Moreover, recent transcriptomic and functional analyses using Seahorse technology have demonstrated that PRRSV-2 infection downregulates genes involved in oxidative phosphorylation in PAMs, leading to drastically reduced mitochondrial respiration and decreased mitochondrial fitness [23]. Notably, ROS elevation was not consistently observed in some of these studies, suggesting that the mechanisms of mitochondrial dysfunction and the resulting inflammatory signals may be context- and strain-dependent [23]. Future studies should systematically examine how different PRRSV strains, including HP-PRRSV versus classical strains, vary in their capacity to induce mtdsRNA release and whether mitophagy serves to limit or exacerbate mtdsRNA-mediated inflammation.

The dual role of PRRSV-encoded nonstructural proteins in inflammasome regulation is particularly noteworthy. Our results confirm that nsp11 robustly suppresses NLRP3 activation by inhibiting ASC oligomerization, consistent with previous screening data [26]. However, recent studies have revealed that other nsps, including nsp2 and nsp5, actively promote NLRP3 activation [27, 28]. In the case of nsp2, the mechanism involves IKKβ-dependent translocation of NLRP3 to dispersed trans-Golgi network compartments, where oligomerization occurs. For nsp5, a single viral protein orchestrates a remarkable “dual immune disruption” strategy: it recruits NLRP3 to the ER–mitochondrial interface, triggering ER stress and Ca^2+^ release to activate the inflammasome; simultaneously, it retains STING in the ER, preventing its trafficking to the Golgi and thereby suppressing type I interferon production [28]. The net effect of these opposing actions—activation of inflammatory cytokines and suppression of antiviral type I interferons—is the “high inflammation, low interferon” phenotype characteristic of severe PRRSV infection. Our demonstration that nsp11 tempers the inflammatory arm of this response by blocking ASC oligomerization suggests that the virus employs a finely tuned strategy: proinflammatory nsps (nsp2, nsp5) drive NLRP3 activation, promoting IL-1β release, while nsp11 simultaneously limits the magnitude of this response to prevent excessive immunopathology that could harm the host. The balance between these opposing nsps likely determines the severity of clinical disease. Although nsp10 also exhibited a comparable suppressive effect in the initial screen, we chose to focus on nsp11 for subsequent mechanistic studies because its known NendoU endoribonuclease activity and the availability of specific pharmacological inhibitors (e.g., A8-A2) provide tractable tools for functional validation and therapeutic exploration. In contrast, the mechanistic basis of nsp10 mediated inhibition remains less defined and warrants separate investigation in future studies.

The regulatory control of nsp11 has recently been illuminated by the discovery that it undergoes K48-linked polyubiquitination at its catalytic residue, K173, mediated by the host E3 ligase TRIM29, leading to proteasomal degradation [30]. This ubiquitination mechanism, which is evolutionarily conserved across most arteriviruses, counteracts nsp11’s immune-suppressive effects and restores interferon production, thereby acting as a host restriction factor [30]. Remarkably, the same catalytic residue targeted for ubiquitination resides within the NendoU active site, raising the possibility that ubiquitination not only marks nsp11 for degradation but may also directly impair its endoribonuclease activity. This intricate regulatory interplay between viral immune evasion and host countermeasures has significant therapeutic implications: small molecules that inhibit NendoU activity, such as A8-A2, have been shown to reduce PRRSV replication and restore interferon responses, indicating that pharmacological targeting of nsp11 could simultaneously suppress viral replication and reverse immune suppression [8]. Future studies should investigate whether such NendoU inhibitors also influence mtdsRNA-induced NLRP3 activation by modulating the inflammatory environment.

The broader significance of mitochondrial nucleic acids as DAMPs extends beyond the PRRSV pathogenesis. For example, in SARS-CoV-2 infection, mtDNA release and activation of the cGAS-STING contribute to the cytokine storm characteristic of severe coronavirus disease 2019 (COVID-19) [40]. In autoimmune diseases such as Sjögren’s syndrome and systemic lupus erythematosus, elevated levels of mtdsRNA and increased SLIRP expression have been linked to chronic inflammation; notably, suppressing SLIRP alleviates the aberrant immune response [22]. These observations suggest the intriguing possibility that PRRSV-induced mtdsRNA release may represent a common pathogenic mechanism shared among diverse viral infections. Cross-viral comparisons could identify conserved pathways that may serve as therapeutic targets across multiple diseases. Importantly, unlike many human disease contexts, the PRRSV‑PAMs model allows for precise genetic dissection of viral and host factors in primary target cells, providing a clear experimental framework that complements cross‑viral observations.

The temporal kinetics of IL‑1β secretion further support a model in which the virus actively balances proinflammatory signaling and immune evasion. As shown in Figure 1A, IL‑1β secretion peaks at 48 hpi and remains elevated through the later stages of infection. Interestingly, this peak coincides with the time when PRRSV nonstructural proteins, particularly nsp11, have accumulated to substantial levels following viral replication. Given our finding that nsp11 inhibits ASC oligomerization, we propose a temporal kinetic model: in the early‑to‑mid phase of infection (approximately 12–36 hpi), mtdsRNA release from damaged mitochondria predominates, driving robust NLRP3 inflammasome assembly and IL‑1β secretion. Then, as infection progresses into the later phase (36–48 hpi), accumulated nsp11 acts as a negative feedback “brake” by blocking ASC oligomerization, thereby tempering the inflammatory response and limiting potential immunopathology. This temporal fine‑tuning likely reflects an evolved viral strategy that balances the benefit of early inflammation for viral dissemination against the cost of excessive tissue damage, which could compromise host survival and ultimately impede viral transmission.

Several limitations of this study should be acknowledged. First, all experiments were performed in primary PAMs or HEK293T cell reconstitution systems; in vivo validation in PRRSV-infected pigs is necessary to confirm that mtdsRNA release and nsp11-mediated ASC inhibition occur in the natural host. Second, although we demonstrated mtdsRNA release using RNAscope, the precise molecular pathways through which mtdsRNA exits damaged mitochondria—via BAX/BAK pores, VDAC oligomers, or other permeabilization mechanisms—remain to be elucidated. Third, the identity of the cytosolic dsRNA sensor(s) that detect mtdsRNA and relay signals to activate the NLRP3 inflammasome in PAMs—whether RIG-I, MDA5, PKR, or another sensor-requires systematic investigation [36–38]. For instance, RIG‑I and MDA5 typically signal through the mitochondrial adaptor MAVS, which has been shown to promote NLRP3 activation via mitochondrial ROS generation or direct protein–protein interactions; PKR, conversely, may modulate inflammasome activity through eIF2α phosphorylation‑dependent translational control or by directly interacting with NLRP3. Whether these distinct sensors converge on common downstream events, such as potassium efflux or mitochondrial permeability transition, remains unresolved. Systematic knockdown or pharmacological inhibition of each candidate in PAMs will be essential to define the dominant pathway linking mtdsRNA sensing to ASC oligomerization. Fourth, given the wide variation in virulence and tissue tropism among PRRSV strains, it is important to compare mtdsRNA release and nsp11 function across different PRRSV-1 and PRRSV-2 lineages. Finally, crosstalk between mtDNA- and mtdsRNA-sensing pathways warrants further investigation, as these mitochondrial DAMPs may act synergistically to amplify inflammatory responses.

## Data Availability

No datasets were generated or analyzed during the current study.
